# Comprehensive Review of Uterine Leiomyosarcoma: Pathogenesis, Diagnosis, Prognosis, and Targeted Therapy

**DOI:** 10.3390/cells13131106

**Published:** 2024-06-26

**Authors:** Qiwei Yang, Obianuju Sandra Madueke-Laveaux, Han Cun, Marta Wlodarczyk, Natalia Garcia, Katia Candido Carvalho, Ayman Al-Hendy

**Affiliations:** 1 Department of Obstetrics and Gynecology, University of Chicago, Chicago, IL 60637, USA; slaveaux@bsd.uchicago.edu (O.S.M.-L.); han.cun@bsd.uchicago.edu (H.C.); aalhendy@bsd.uchicago.edu (A.A.-H.); 2 Department of Biochemistry and Pharmacogenomics, Faculty of Pharmacy, Medical University of Warsaw, Banacha 1B, 02-097 Warsaw, Poland; marta.wlodarczyk@wum.edu.pl; 3 Greehey Children’s Cancer Research Institute, University of Texas Health Science Center, San Antonio, TX 78229, USA; natalia.garciaft@gmail.com; 4 Department of Cell Systems and Anatomy, University of Texas Health Science Center, San Antonio, TX 78229, USA; 5 Laboratório de Ginecologia Estrutural e Molecular (LIM 58), Disciplina de Ginecologia, Departamento deObstetricia e Ginecologia, Hospital das Clinicas da Faculdade de Medicina da Universidade de Sao Paulo (HCFMUSP), São Paulo 05403-010, Brazil; carvalhokc@gmail.com

**Keywords:** uterine leiomyosarcoma, leiomyoma, uterine fibroids, sarcoma, diagnostics, prognosis, distinguishment, biological pathways, epigenetics, epitranscriptome, targeted therapy

## Abstract

Uterine leiomyosarcoma (uLMS) is the most common subtype of uterine sarcomas. They have a poor prognosis with high rates of recurrence and metastasis. The five-year survival for uLMS patients is between 25 and 76%, with survival rates approaching 10–15% for patients with metastatic disease at the initial diagnosis. Accumulating evidence suggests that several biological pathways are involved in uLMS pathogenesis. Notably, drugs that block abnormal functions of these pathways remarkably improve survival in uLMS patients. However, due to chemotherapy resistance, there remains a need for novel drugs that can target these pathways effectively. In this review article, we provide an overview of the recent progress in ascertaining the biological functions and regulatory mechanisms in uLMS from the perspective of aberrant biological pathways, including DNA repair, immune checkpoint blockade, protein kinase and intracellular signaling pathways, and the hedgehog pathway. We review the emerging role of epigenetics and epitranscriptome in the pathogenesis of uLMS. In addition, we discuss serum markers, artificial intelligence (AI) combined with machine learning, shear wave elastography, current management and medical treatment options, and ongoing clinical trials for patients with uLMS. Comprehensive, integrated, and deeper insights into the pathobiology and underlying molecular mechanisms of uLMS will help develop novel strategies to treat patients with this aggressive tumor.

## 1. Introduction: Biology and Pathogenesis of Uterine Leiomyosarcoma 

Uterine leiomyosarcoma (uLMS) is the most common type of uterine sarcoma. It is an aggressive tumor associated with high recurrence rates, metastasis, and poor prognosis, with a 5-year survival rate of 10 to 15% when the initial diagnosis is with metastatic disease [1]. Currently, transformative therapeutic options in uLMS are limited, and there is an urgent need to develop novel strategies to improve outcomes for uLMS patients. So far, chemotherapy as an adjunct to surgery has gained much more attention and anticipation, according to the clinical characteristics and recurrence risks [2]. Doxorubicin and trabectedin are already approved for sarcoma and are available as generic drugs. Prognostication is a crucial issue for sarcoma patients’ care as it triggers chemotherapy application. The Complexity Index in SARComas (CINSARC) is a transcriptomic signature related to identifying high-risk sarcoma and high metastatic potential. Consequently, CINSARC is currently evaluated in clinical trials for treatment response prediction and stratification of therapy [3]. The CINSARC signature with the genomic index can potentially improve diagnoses, therapeutic strategies, and randomization in future clinical trials [4]. Epigenetic drugs have shown great promise in pre-clinical models of cancers. However, the emerging clinical results of many epigenetic therapies do not directly translate into the clinical arena because resistance to these drugs rapidly emerges. Therefore, if epigenetic drugs are capable of contributing effectively to the clinical management of patients with uLMS, they must be integrated into rational combination regimens that work synergistically with these drugs. Therefore, there is an unmet need to develop novel strategies for treating patients with uLMS. In this review article, we provide the current state of knowledge by summarizing and discussing the cellular and molecular mechanisms underlying the regulation and emerging role of genetic, epigenetic, and biological pathways in the pathogenesis of uLMS. We also discuss applications that can be exploited to enhance the therapeutic efficacy of uLMS. 

## 2. Differential Diagnosis of Uterine Leiomyosarcoma and Leiomyoma

### 2.1. Challenges with Differentiating Uterine Leiomyoma from Uterine Leiomyosarcoma

Uterine leiomyoma (LM) and uLMS are considered biologically different tumors. However, these two tumors share some characteristics that make their differentiation using current clinical diagnostic tests challenging. In contrast to malignant uLMS, LMs are benign neoplasms of the myometrium, representing the most common tumors globally in women of reproductive age [5]. Notably, uLMS and LM share several common clinicopathological and imaging features that complicate their differential diagnosis [6] (Table 1). Similar clinical presentations shared by LM and uLMS include heavy and abnormal menstruation, anemia, pelvic pressure, and bulk symptoms (urinary or bowel symptoms) [7]. The management of each diagnosis is vastly different. LMs are benign and are treated based on the degree and severity of symptomatology, while uLMS have a high malignant potential and require immediate invasive surgical treatment, i.e., hysterectomy and adjuvant chemotherapy. This dichotomy between similar clinical presentations and starkly different management strategies poses a significant clinical dilemma for physicians and affected patients. This dilemma is further complicated by the inability to differentiate between uLMS and LM reliably prior to surgical resection and pathologic evaluation [8]. Consequently, patients are at risk of receiving delayed and inappropriate treatment, i.e., unnecessary invasive, high morbidity, and non-fertility-sparing surgical treatment for benign LM and conservative, less invasive surgery for uLMS with resultant iatrogenic upstaging of malignancy, lower disease-free survival, and poorer overall survival (OS) (10.8 months vs. 39.6 months) [9]. 

Currently, the diagnosis of uLMS is established at the time of pathologic analysis of the resected tumor. This method remains the gold standard [8]. Accordingly, the differential and conclusive diagnosis between benign and malignant uterine tumors is exclusively post-surgical and based on histological examination [10]. In histologic evaluations, conventional uLMS appear hypercellular with severe nuclear atypia, tumor cell necrosis, and high mitotic rate (≥10 mitoses figures per 10 high power fields). However, the diagnosis can be made depending on the presence of at least two of these criteria [11]. 

An epithelioid tumor is a variant containing more than 50% epithelioid appearance cells, consisting of polygonal cells showing eosinophilic or clear cytoplasm arranged in nested, corded, nodular, or diffuse patterns. The myxoid uLMS contains abundant myxoid stroma and may display vague fascicular or nodular growth [12]. Both epithelioid and myxoid uLMS usually show mild nuclear atypia, and their mitotic rate often represents less than 3 mitotic figures/10 HPFs. In epithelioid tumors, necrosis may be absent, while myxoid ones are often hypocellular. In the absence of severe cytologic atypia and high mitotic activity, those tumors may be diagnosed as sarcomas based only on their infiltrative edges [11,13,14,15]. 

Unfortunately, however, some features of uLMS are not seen in myxoid and epithelioid variants of LMS. These variants, although rare, are very aggressive and may feature mild atypia, absence of necrosis, and a low mitotic rate. Their cytologic features also vary from conventional uLMS. They can thus pose a diagnostic challenge [16] (Table 2). Additionally, uLMs usually express smooth muscle markers such as desmin, h-caldesmon, smooth muscle actin, and histone deacetylase 8 (HDCA8). However, epithelioid and myxoid uLMS present a lack or reduced amount of those markers. Moreover, uLMS can be positive for CD10, epithelial markers (keratins and EMA), estrogen, progesterone, and androgen receptors.

In recent years, much effort has been made to develop preoperative diagnostic tests for the evaluation of suspicious uterine tumors. Each test has varying degrees and ranges of accuracy—endometrial biopsy, 58–64% [17,18]; pelvic ultrasonography, 11–56%; pelvic MRI, 35.3–80% [19,20]; lactate dehydrogenase (LDH); and sensitivity, 0.47 [21]. The use of these tests in combination with patient demographics, such as age and menopausal status, has been proposed for use independently or as part of an algorithm to determine a “preoperative risk score” for uterine tumors [7,21,22,23]. Although some of these evaluative tools show promise, their reported testing properties must be interpreted cautiously based on the generalizability of the studies to the population at large [24]. Thus, despite the progress made so far, the preoperative diagnosis of LMS remains a challenge, and improving accurate diagnosis through novel and innovative approaches remains a critical and unmet need.

### 2.2. Comparison between Uterine Leiomyosarcoma and Leiomyoma

#### 2.2.1. Clinicopathologic Features

LM and uLMS share several common clinicopathological features. This similarity in clinical presentation can lead to a diagnostic challenge between these two uterine tumors. Clinically, both uterine tumors present primarily with abnormal, heavy, and prolonged uterine bleeding, pelvic pain/pressure manifesting as bowel or bladder function difficulties, and the presence of a pelvic mass [25,26]. Both tumors arise from the myometrium or muscle wall layer of the uterus, as focal masses, and may appear similarly in imaging such as a pelvic ultrasound, CT scan, and magnetic resonance imaging (MRI). Additionally, uLMS and LM share morphological and molecular characteristics that cannot be differentiated through current clinical diagnostic tests (Table 1). As previously discussed, despite many efforts to design preoperative risk scores and biomarkers to diagnose uLMS preoperatively, there are no reliable clinical diagnostic tools to achieve this goal. Finally, the epidemiological and public health literature confirms that Black women are at higher risk for both LM and uLMS [27,28]. LMs are four times more prevalent and cause more debilitating symptoms in Black women at earlier ages than their non-Black counterparts. Black women are twice as likely to have uLMS and have lower survival rates and higher mortality rates with the diagnosis of uLMS [29]. Unlike uLMS, LM does not develop or grow in the post-menopausal phase without hormonal therapy. Clinical scenarios such as this should prompt suspicion of a uLMS [30]. 

A rare benign variant of LM that is often feared to be malignant is benign metastasizing LM (BML). BML occurs frequently in reproductive or premenopausal women with a surgical history of uterine myomectomy or hysterectomy. These tumors originate from smooth muscle cells and then metastasize to extrauterine sites, including the lungs (80%), heart, bones, liver, and central nervous system. They rarely metastasize to lymph nodes, although these have been reported. The treatment of BML is not standardized, but resection of tumors with hormone suppression is commonly performed [31]. 

Uterine smooth muscle tumors of uncertain malignant potential (STUMPs) are a poorly defined subcategory of uterine smooth muscle tumors, separate from benign LM and malignant LMS. They were first described by Kempson et al. in 1973 and further characterized by cytologic atypia, mitotic count, and tumor cell necrosis [32,33]. The clinical presentation is similar to uterine LM and LMS with abnormal uterine bleeding, pelvic pain or pressure, or uterine mass noted on an exam or imaging. They are only diagnosed by pathologic evaluation, with limited use of preoperative imaging. MRI with LDH level, PET scan, and ultrasonography have been proposed as tools to distinguish between STUMPs and malignant lesions, but these are nondiagnostic with limited use [34]. The World Health Organization (WHO) defines a STUMP as a smooth muscle tumor with features that preclude an unequivocal diagnosis of LMS, however, it does not fulfill the criteria for a benign LM or its variants [35]. The tumor must exhibit one of the following histologic criteria: (I) tumor necrosis in atypical leiomyoma; (II) necrosis of uncertain type with >10 mitotic figures per 10 high power fields (HPFs) or severe diffuse atypia; (III) severe diffuse or focal atypia with borderline mitotic counts; or (IV) necrosis difficult to categorize. The risk of relapse is variable but up to 36.4% [34]. The overall prognosis is favorable with a 5-year disease-free survival of 66% and 5-year overall survival of 92% [36]. Recurrence may occur in the form of STUMP or LMS [34]. Reported sites of recurrence include the pelvis, chest, bone, and brain [36,37,38,39]. Due to its rarity, there have been no correlations found between recurrence rate and patient age, ethnicity, smoking habit, and type of surgery [37]. The treatment of choice for recurrent disease is surgical resection if feasible. 

#### 2.2.2. Tissue Origin and Driver Mutations

LMs are smooth muscle tumors of monoclonal origin. They arise from the smooth muscle wall of the uterus [40]. It is hypothesized and demonstrated that LMs originate from the abnormal stem cells of the myometrium [41,42,43,44]. However, the specific cell of origin has yet to be identified [45]. Advanced genomic technologies and functional studies have demonstrated that genetic alterations are responsible for LM formation. *MED12* mutations are the most prevalent mutations occurring in up to 90% of LM depending on patient ethnicity [46]. The overexpression of *HMGA2* has also been implicated in the pathogenesis of a proportionally smaller subset of LMs [47].

Evidence suggests that uLMS can arise de novo or secondary to malignant degeneration of an LM. uLMS is a malignant mesenchymal neoplasm that arises from the myometrium of the uterus. The cells of uLMS show distinct features of smooth muscle and, on histology, appear as intersecting spindle cells with eosinophilic cytoplasm and elongated and hyperchromatic nuclei [48]. This heterogeneity complicates the identification of driver mutations and therapeutic targets. No single driving mutation has been identified for uLMS, but some potential driving mutations have been reported [49]. uLMSs have complex karyotypes and aneuploids, which are not commonly seen in benign tumors, including LMs, suggesting that uLMS is likely a distinct entity from LM. However, microarray data have identified a subset of LMs that exhibit deletions and share transcriptomic profiles that cluster with those of uLMSs, suggesting that malignant degeneration of a subset of LMs may occur [50]. The Cancer Genome Atlas project confirmed mutations and deletions in *RB1*, *p53*, and *PTEN* in all LMS, including uLMSs, and an overall low mutational burden in sarcomas compared to other tumors. 

#### 2.2.3. Morphology

**Figure 1:**
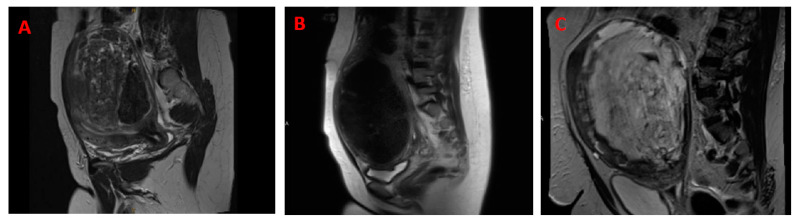
Reproductive-aged women with a large pelvic mass. Both presented with heavy menses, pelvic mass, and pressure. An MRI was obtained to better visualize large masses that were identified on the ultrasound. (**A**) A 45-year-old with a large pelvic mass. MRI: possible myxoid though features overlap with leiomyosarcoma. (**B**) A 44-year-old with a uterine mass. MRI: dominant anterior mass. Leiomyosarcoma cannot be entirely excluded. Pathology confirmed: benign leiomyoma. (**C**) A 38-year-old who presented with a pelvic mass and abnormal uterine bleeding; MRI sagittal view of a patient who presented with a pelvic mass and abnormal uterine bleeding.

When confined to the uterus, uLMSs present as solitary, large, and often palpable intra-myometrial tumors in more than half of cases. Similarly, although they more commonly exist in multiples, LMs may present as a solitary palpable mass [40]. Another overlap between the two tumors is that 40 to 50% of uLMSs, like LM, express progesterone, estrogen, and androgen receptors. Beyond these similarities, the two tumors do not share much more (Table 1). The diagnostic challenge between uLMS and LM exists when LMs fail to have a classic appearance on imaging. That classic appearance may be described as a well-circumscribed tumor with a homogenous, hypoechoic appearance on ultrasound or homogenous T2 hypointensity and T1 isointensity relative to the outer myometrium [51]. LMs, classically, have a distinct whorled appearance while uLMS tumors have a softer consistency. When LMs are atypical in appearance or have undergone a form of degeneration, there begins to be more of an overlap in the appearance of the two tumors. For example, the LM described as having higher cellularity than normal myometrium demonstrates a softer texture than classic LM [51]. However, in histologic examinations, there is no evidence of atypia or necrosis. Another overlap can be seen with myxoid degeneration of LMs. This degeneration is uncommon and has a large volume of extracellular mucin. Myxoid features (Figure 1) can be seen in uLMS. However, unlike LM in which a well-demarcated border will be retained, uLMSs have a more infiltrative appearance [51].

Although very large LMs may undergo necrotic or cystic degeneration due to an outgrowth of blood supply, microscopically, up to 80% of uLMSs distinctly contain extensive areas of tumor cell necrosis and hemorrhage, hypercellularity, and severely atypical nuclei with >15 mitotic figures per 10 high power fields (HPFs) [51,52]. It is important to highlight that the infarct-type necrosis seen in benign lesions is distinct from the tumor necrosis seen in uLMSs. Lastly, compared to LMs, uLMSs lack a sharply demarcated or defined border and invade adjacent myometrium [30].

#### 2.2.4. Behavior, Onset, and Incidence

uLMS is a rare and malignant tumor that represents ~1% of all uterine soft tissue malignancies. It originates from the smooth muscle wall of the uterus and behaves aggressively. The prognosis of uLMS is the worst of all soft-tissue sarcomas with the lowest survival rate. The average age of patients with this diagnosis ranges from 40 to 50 years old, and approximately 6 out of 1000 patients are diagnosed annually [53]. Common metastatic sites for uLMS include the lungs, liver, brain, kidney, and bones [54].

Unfortunately, uLMS shares many common clinical grounds with LM. In addition to originating from the smooth muscle layer of the uterus, both tumors present with clinical symptoms of heavy menses, pelvic mass, and pelvic pressure/pain (Table 1). The age of incidence for both tumors overlaps substantially with the only distinct exception of tumor growth in the post-menopausal phase in a patient who is not on hormonal therapy. This clinical setting should prompt suspicion [30]. Beyond this specific scenario, LM and uLMS can occur in patients with similar demographics. Specifically, both tumors are more common in Black women and with increasing age. Several studies report that women aged 41–60 were significantly more likely to have a fibroid diagnosis compared to those <age 30 [55]. The silver lining in the diagnostic dilemma between uLMS and LM is that LMs are non-cancerous tumors and mortality from this condition is extremely rare. However, LMs are the most common neoplasm in women of reproductive age, and despite their non-malignant nature, LMs can significantly impact affected patients’ quality of life, causing severe symptoms in approximately 25% of patients. LMs are the leading cause of hospitalization for gynecologic disorders and remain the most common indication for hysterectomy in the United States [56]. 

#### 2.2.5. Genetic Mutation, Molecular Prognostic Biomarkers, and Transcriptional Difference

The genome-wide DNA sequencing approaches have been employed to determine the genetic difference between uLMS and LM (Table 1). The whole-exosome sequencing comparison analysis in 44 LM and 34 uLMS tumors revealed higher tumor mutations in uLMS than in LM, including single-nucleotide variants, indels, and copy number variants [57]. A study by Nasioudis et al. demonstrated that a high incidence of *TP53* (67%), *RB1* (42%), *PTEN* (16%), and *MED12* (13%) were observed in a large cohort of uLMS (*n* = 325). In addition, 6% of gene mutations for *CDKN2A* and *CDKN2B* were also identified from the same cohort samples [58]. These mutations are rarely observed in benign LM except for the *MED12* mutation, which exhibited a much higher frequency (~80%) in LM. In a separate study, *ATRX*, *TP53*, and *PTEN* showed top somatic mutations in uLMS [59]. These gene mutations define the genetic landscape of uLMS and may provide potential targeted drugs for LMS treatment.

In addition to histopathological and clinical factors, molecular risk stratification as a molecular criterion should be considered in clinical practice [60,61]. Recently, a genomic risk stratification model for a cohort of 238 uLMSs was reported [62]. For both disease-specific survival (DSS) and progression-free survival (PFS) in these uLMS cohort samples, tumor size (>20 cm vs. 10–20 cm vs. <10 cm), mitotic rate >10 per 10 HPFs, and the presence of necrosis were significantly associated with worse outcome. A 3-tier genomic risk group is proposed for DSS and PFS, including high risk (*TP53* Mutation + *ATRX* Mutation, or *TP53* Mutation + chr20q amplification), intermediate risk (*TP53* Mutation or *ATRX* Mutation or chr20q amplification), and low risk (lack of any of these three alterations). This 3-tier genomic risk stratification was associated with DSS and PFS, suggesting that different sets of mutations were associated with inferior survival in uLMS [62].

The transcriptome analysis revealed a distinct expression pattern between benign LM and malignant LMS. The RNA-seq analysis identified 489 differentially expressed genes between the two types of tumors associated with enriched pathways of cell cycle-associated processes, such as chromosome segregation, DNA replication, meiotic cell cycle, and G2/M phase transition [57]. 

Currently, pathological biopsy is the gold standard for uLMS diagnosis. However, LM and uLMS share some common characteristics, which makes the preoperative distinguishing of these two tumors challenging. Myxoid and epithelioid LMS, BML, and STUMP variants provide additional complexity for diagnosing uterine tumors. At molecular levels, uLMS shows different genetic and transcriptome patterns compared to LM, which offers distinct molecular signatures for this malignant tumor. 

## 3. Role of Biological Pathways in uLMS 

### 3.1. DNA Repair Pathways

**Figure 2:**
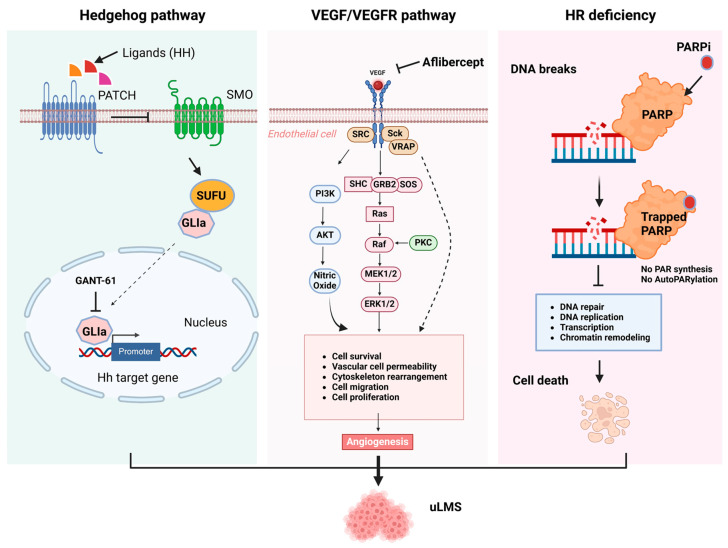
Abnormal signaling pathways contribute to the pathogenesis of uLMS. Hedgehog pathway (**left panel**): The signaling pathway is activated by three hedgehog proteins (Hh): sonic hedgehog (Shh), Indian hedgehog (Ihh), and desert hedgehog (Dhh). Hh binds and inactivates a transmembrane protein PATCH. After the binding of Hh, PATCH is sent into the cell and degraded by the proteasome. The PATCH degradation activates Gli proteins, which translocate into the nucleus and switch on specific gene expressions. uLMS showed activated HH signaling. In vitro and in vivo studies demonstrated that targeting GLI with its inhibitor suppressed the uLMS growth. VEGF/VEGFR (**middle panel**): VEGF binds to the VEGF receptor and triggers the activation of downstream signaling, causing cell survival, vascular cell permeability, cytoskeleton rearrangement, cell migration, and cell proliferation. Targeting VEGF with aflibercept inhibited the uLMS growth. Homogenous recombination (**right panel**): HR comprises a series of interrelated pathways that repair DNA breaks and interstrand crosslinks. uLMS harbors frequent somatic homozygous *BRCA2* deletion, leading to HR deficiency. PARPi competes with NAD^+^ at the catalytic domain of PARP to block PARP catalytic activity and the formation of PAR polymers, which suppress the DNA replication, DNA repair, chromatin remodeling, and gene transcription in the presence of BRCA 1/2 mutations, eventually causing cell death.

Homologous recombination (HR) is widely used by cells to accurately repair damaged DNA breaks that occur on both strands of DNA, known as double-strand breaks (DSBs), in a process called homologous recombinational repair [63]. HR comprises a series of interrelated pathways that function in repairing DNA double-stranded breaks (DSBs) and interstrand crosslinks (ICLs) and play a critical role in maintaining genomic stability (Figure 2). Uterine sarcoma patients in a relatively large cohort sample (*n* = 145) showed frequently harbored pathogenic alterations in HR-DNA damage repair (DDR)-related genes [58]. uLMS typically has complex karyotypes, aneuploids, and many other genetic defects suggesting dysfunction of DNA repair pathways, including HR in LMS. Indeed, LMS exhibits a significantly higher rate of HR pathway alterations compared with other subtypes of soft-tissue sarcomas (STSs) [64]. Some HR-DDR gene mutations are prevalent in uLMS, impacting the DNA repair capacity in uLMS. *ATRX* gene plays a critical role in HR-DDR and maintaining telomere stability. ATRX, in collaboration with the DAXX protein, facilitates the binding of the H3.3 histone to the telemetric and pericentromeric chromatin. ATRX is critical in HR-DDR and protects stalled replication forks from degradation. ATRX also cooperates with FANCD2 to promote HR-dependent repair of dsDNA breaks. A loss of ATRX is associated with the activation of alternative lengthening of telomere pathways in tumors and impacts the HR repair signaling [65]. Patients with ATRX/DAXX mutations were characterized by a significantly shorter survival, supporting the view that alterations in this DNA repair pathway trigger a more aggressive uLMS phenotype [59]. Several studies revealed a high incidence of ATRX mutation (20–51%) among uLMS patients [59,66]. Studies reveal that LMS harbors defects in components of the HR repair pathways and is sensitive to treatments that induce double-stranded breaks or replication fork arrest. Notably, in a preclinical model, Elimusertib (BAY1895344), an ATR inhibitor, showed in vivo activity against ATRX-mutated uLMS patient-derived xenografts [67]. Alterations in BRCA1/2 were significantly more common in uLMS compared with non-uLMS [64], and LMS patients with BRCA1/2 loss trended towards a more poorly differentiated histologic subtype as well as an increased mitotic count, highlighting the connection between HR and cancer predisposition. Whole genome sequencing and whole exome sequencing analyses demonstrated a deletion in BRCA2 in the uLMS tumors [68]. HR comprises a series of interrelated pathways that repair DNA double-stranded breaks and interstrand crosslinks. PARP-inhibitor therapy improved patient outcomes in BRCA2-deleted uLMS [68].

### 3.2. Hedgehog Pathway

The hedgehog (HH) pathway is a conserved evolutionary signaling pathway that controls embryonic development and regulates differentiation, physiological conditions, and normal cell growth. It also plays an essential role in maintaining adult stem cells [69,70]. The deregulation of the HH signaling pathway has been linked to several human disorders, including cancer [71,72,73,74,75,76,77,78]. Three proteins, including the HH ligand, Patched (Ptch), and SMO, are involved in the activation of HH signaling [79,80]. The HH pathway can be activated by two different mechanisms: canonical or non-canonical [81,82]. Three ligands, including sonic hedgehog (Shh), Indian hedgehog (Ihh), and desert hedgehog (Dhh), can activate the HH signaling pathway. The activation of the HH pathway by a canonical mechanism involves the primary cilium with its specialized transmembrane receptors. The HH ligand binds to the Ptch1 receptor, causing the accumulation of SMO protein in the primary cilium, which signals the SUFU (suppressor of fused) to release GLI proteins [83]. The GLI activators (GLIa), mainly GLI1, are translocated into the nucleus to activate the transcription of HH target genes [83,84,85,86]. However, in some situations, GLI can be activated via a non-canonical HH signaling mechanism, independent of ligand and SMO involvement [82,86]. 

Abnormal expression of key members in HH pathways has been discovered, and targeting SMO and GLI1 has been demonstrated to exert an anticancer effect in vitro and in vivo in different types of cancer [87,88]. The HH pathway was described for the first time in uLMS in 2016 by Garcia et al. [72], showing that uLMS patients presented higher protein levels of SMO and GLI1 compared to LM and normal myometrium [72]. We have tested the efficacy of SMO and GLI inhibitors alone or in combination with a DNA methyltransferase inhibitor in uLMS cells, showing a decrease in LMS proliferation, migration, and invasion (Figure 2). The FDA has approved several SMO inhibitors and tested them in clinical trials, showing promising results. We investigated the effect of the GLI1 inhibitor in an LMS xenograft animal model and demonstrated a suppressive effect of GLI1 inhibition on LMS growth in vivo [76]. The regulation of the HH signaling pathway in LMS has been correlated with the expression of the transcription factor NKX6-1. NKX6-1 acts through the SHH pathway to increase cell proliferation, drug resistance, cancer stemness in vitro, and tumorigenicity in vivo. An SHH inhibitor (RU-SKI 43), which suppresses the SHH acyltransferase HHAT, showed a reduction in cell survival in NKX6-1 expressing cells, indicating that an SHH inhibitor could be useful for uLMS treatment [89]. Therefore, targeting the HH signaling pathway in LMS may open a new potential therapeutic target for treating this aggressive tumor. 

### 3.3. VEGF/VEGFR

Angiogenesis is a fundamental biological process of new capillaries forming out of the preexisting blood vessels, required for many biological events, including embryonic development, tissue repair, ovulation, and menstruation. In physiological angiogenesis, this process is tightly regulated by pro- and anti-angiogenic factors and provides oxygen-rich blood to organs and tissues. However, under pathological conditions, such as cancer, the dysregulation of pro- and anti-angiogenic factors can trigger angiogenesis and support tumor growth and metastasis feeding tumors with nutrients and oxygen [90,91]. Among them, aberrant VEGF signaling has been recognized as the key regulator in tumor angiogenesis. VEGF signaling is induced by binding VEGF ligands to their cognate membrane-bound receptors, activating multiple downstream pathways, including PI3K/AKT and Ras/MAPK pathways. Targeted inhibition of the VEGF pathway has demonstrated efficacy in many types of tumors. In uLMS and other soft tissue sarcomas, VEGF/VEGFR signaling is associated with an increased risk of progressive disease and patient survival [92]. Aflibercept, VEGF Trap, is a recombinant fusion protein combining VEGFR receptors 1 and 2 domains with the IgG1 Fc fragment (Figure 2). It is a potent angiogenesis inhibitor, acting as a high-affinity soluble decoy VEGFR receptor in a phase II clinical trial. Anti-VEGF targeted treatment with aflibercept showed modest activity in patients with uLMS [93]. Moreover, the anti-VEGFR inhibitor combined with other inhibitors, such as doxorubicin and pembrolizumab, has been investigated [92,94]. Therefore, targeting angiogenesis is an attractive therapeutic option in uterine sarcoma. 

### 3.4. Immune Checkpoint Blockade 

Immune checkpoint blockade (ICB) has emerged as an up-and-coming therapeutic option for patients with several types of cancers [95]. The immune cell composition in uLMS consists of several immunosuppressive cell populations, including regulatory T cells (Tregs), myeloid-derived suppressor cells (MDSCs), and tumor-associated macrophages (TAMs) [12,14,21]. These immunosuppressive cells are critical for conferring resistance to ICB [22] and mediating immune escape from cancers [96]. Evidence indicates that tumor-intrinsic pathways, including the PI3K/mTOR pathway, Wnt/β-catenin pathway, VEGF and VEGFR tyrosine kinases, are critical in regulating the immune response. A study on 21 cases using high-dimensional analysis revealed the adaptive and innate immune cell landscape of uLMS. The analysis showed heterogeneous characteristics of uLMS patients, particularly on the infiltrating macrophages [97].

PD-1 (programmed cell death protein 1) and PD-L1 (programmed death ligand 1) inhibitors act to inhibit the association of PD-L1 with its receptor, PD-1. The interaction of these cell surface proteins is involved in suppressing the immune system. It occurs following infection to limit the killing of bystander host cells and prevent autoimmune diseases. Since the role of PD1/PDL-1 was identified as a cancer treatment target in the immune system, targeted inhibition of PD-1 and PDL-1 has been used in multiple cancers. In uLMS, immunotherapy with PD-L1 inhibition has been explored in a phase II study. Nivolumab administration as a monotherapy did not show any benefit or response [98]. Combination therapy with an anti-VEGFR tyrosine kinase inhibitor (axitinib) and an immune checkpoint inhibitor (pembrolizumab) showed preliminary activity and response in patients with advanced sarcoma [92]. 

### 3.5. Protein Kinases and Intracellular Signaling Pathways 

The protein kinases are classified into two main families based on the ability to phosphorylate either serine and threonine or tyrosine residues. The latter consisted of receptor and non-receptor types. Protein kinases execute their biological functions by controlling a variety of cellular responses, including cell division, metabolism, migration, cell–cell interaction, cell survival, and apoptosis [99]. Receptor tyrosine kinases (RTKs) act as receptors for a wide range of external signals that regulate many biological processes, and play a pivotal role in disease progression, including tumorigenesis. RTKs contain three domains: extracellular receptor, transmembrane, and cytoplasmic domain. Transcriptome analysis revealed that the expression of several cell cycle-related kinases was upregulated in uLMS compared to LM [100], suggesting that the pathological deregulation of these protein kinases is implicated in the occurrence of tumors. The inhibition of PLK1 and CHEK1 induced cell cycle arrest and caused DNA damage in uLMS cells, and targeted inhibition of PLK1 with BI-2536 exhibited anticancer effects in the preclinical animal model [100]. Although targeted inhibition of CHEK1 with prexasertib monotherapy showed no considerable tumor regression effect, combining prexasertib with cisplatin significantly reduced the tumor volume compared with cisplatin monotherapy [100]. Another study showed that inhibition of endogenous neurotrophic tyrosine receptor kinase (TrkB) signaling by treatment with either the soluble TrkB ectodomain or the Trk receptor inhibitor K252a suppressed uterine sarcoma cell proliferation and increased apoptosis [101]. Moreover, treatment with K252a suppressed tumor growth in athymic nude mice bearing multidrug-resistant uterine sarcoma cell-derived tumors [101]. PI3K/AKT/mTOR signaling plays a vital role in regulating many biological processes via intracellular signaling mechanisms. PI3K activation phosphorylates and activates AKT, which can impact multiple downstream effectors, including mTOR. Phosphorylated S6 is a crucial downstream player in the mTOR pathway, and PTEN inhibits the PI3K pathway. Up to 33% of uLMS cases involve the PI3K/mTOR pathway, which is overactivated due to the loss of function of negative regulators, including PTEN [95,102,103]. Rapamycin or LY294002 (PI3K/AKT/mTOR pathway inhibitors) in combination with HDACis showed a synergistic effect on uterine sarcoma cells [104]. These studies hint towards the critical role of PI3K/AKT/mTOR pathways in the uLMS tumorigenesis. In clinical application, tyrosine kinase inhibitor pazopanib in a retrospective analysis showed a promising effect for sarcoma patients. Most patients with sarcoma had uLMS with a median overall survival (OS) of 17.5 months compared to 11.1 months for the nonuterine population [105,106]. 

### 3.6. Wnt/β-Catenin Pathways

The Wnt/β-catenin signaling pathway is involved in many physiological events, and dysregulation of this pathway is involved in many diseases [107,108]. In contrast to understanding Wnt signaling in benign LM [109,110], the knowledge of the role and regulation of Wnt signaling in uLMS is limited. A study by Kildal et al. reported that 25%, 36%, and 23% of 245 LMS patients showed an upregulation of β-catenin in the membrane, cytoplasm, and nuclei of the primary LMS, respectively. The membranous β-catenin was significantly associated with poor crude survival in univariate, but not in multivariate analyses [111]. Secreted frizzled-related proteins (SFRPs) are modulators of Wnt signaling involved in the pathogenesis of various neoplasms. A separate study demonstrated that the expression levels of SFRP4 in uLMS tissues were lower than those in normal smooth muscle and LM. SFRP4 suppressed viability and migration and enhanced the uLMS cell adhesion [112]. However, further investigation is needed to fully understand the implications of Wnt/β-catenin signaling in uLMS. 

These findings reveal that divergent pathways participate in the uLMS pathogenesis, reflecting the complex mechanisms underlying this aggressive tumor development. Targeted inhibition of crucial pathways with combination treatment options shows great promise for this clinically significant disease.

## 4. The Role of Epigenetics in the Pathogenesis of Leiomyosarcoma

### 4.1. DNA Methylation

DNA methylation is a pivotal epigenetic modification that affects gene expression, and its dysfunction is strongly implicated in cancer development. Global DNA hypomethylation or loss of methylation has been associated with genomic instability, aneuploidy, loss of imprinting, reactivation of transposable elements, and endogenous retrovirus [113]. Cancer cells possess an extensively hypomethylated genome with localized regions of hypermethylation at CpG islands at the promoter and regulatory regions of target genes. 

**Figure 3:**
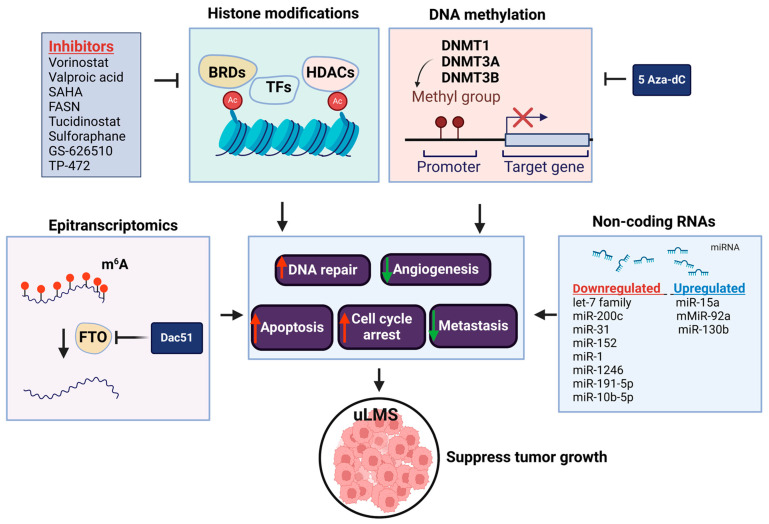
Abnormal epigenetic/epitranscriptomic regulation contributes to the pathogenesis of uLMS. Histone modification reader BRD9, DNA methyltransferases, class I HDACs, and m^6^A eraser (FTO) are upregulated in uLMS compared to MM. The targeted inhibition of BRD9, HDACs, and FTO with their small specific inhibitors can inhibit the LMS growth in vitro via induced apoptosis and cell cycle arrest. Red arrows indicate the increased activity of cellular events; Green arrows indicate the decreased activity of biological events.

DNA methylation profiling studies have been conducted in sarcoma, showing that the analysis of methylation patterns can distinguish different sarcoma subtypes. For instance, uLMS and soft tissue LMS display different methylation signatures [114]. We have compared the expression levels of DNA methyltransferases between uLMS and myometrial cell lines and revealed that the RNA and protein levels of DNMT1 and DNMT3a showed a marked increase in uLMS cells compared to smooth muscle cells. Targeted inhibition of uLMS cells with a DNA methyltransferase inhibitor, 5-aza-dc, suppressed LMS cell phenotype with decreased cell proliferation and migration and increased apoptosis (Figure 3). Notably, the HH pathway is deactivated in response to 5Aza-dc treatment [77]. These results offer various potential avenues for suppressing LMS phenotype and enhancing antitumor activity.

Due to the highly conserved characteristics of cancer-specific methylation, liquid biopsies provide a promising setting for methylation-based biomarker application. The advent of this minimally invasive experimental approach with high-throughput next-generation sequencing and advanced bioinformatics and computational tools will enhance the development of early detection, diagnostics, and prognostics in patients with uLMS [115]. 

### 4.2. Histone Modification

The mechanisms responsible for the remodeling of chromatin structure and, as a result, regulation of gene expression include covalent post-translational modifications of histones, such as methylation, acetylation, phosphorylation, ubiquitination, and SUMOylating of amino-acid residues in N- and C-terminal regions of the histone protein [116,117]. HATs catalyze adding acetyl groups to lysine residues located on the N-terminal tails of histones, resulting in the destabilization of the higher-order helix of the nucleosome [116]. The enzymes that remove the acetyl group from histone proteins and have a repressive effect are histone deacetylases—HDACs [118,119,120]. Additionally, histone methyltransferases (HMTs) catalyze the transfer of the methyl group to the vicinity of histone tails containing arginine or lysine residues. In turn, histone demethylases (HDMs) are responsible for removing methyl groups from these regions [121]. Another modification that histone proteins may undergo is the phosphorylation of serine, threonine, and tyrosine residues located in histone tails, and it is associated with transcription activation [122]. The effect of the histone modification often depends on the site of change. For instance, mono-, di-, and trimethylation of lysine residues in histones H3 and H4 (H3K4, H3K36, H3K79) provide the active form of chromatin results. At the same time, other modification locations (H3K9, H3K27, H4K20) provide a tight form and lead to gene silencing [123]. 

The role and regulatory mechanism of histone modifications have been extensively investigated in uLMS [113,124,125] (Table 3). Abnormal regulations of HDAC family members were identified in LMS compared to normal myometrial tissues [126,127,128]. Data from mass spectrometry imaging showed that the presence of two specific histone H4 variants (annotated at *m*/*z* 11,314 and 11,355) was associated with poor survival of LMS patients [129]. These studies suggested that HDACs may contribute to LMS pathogenesis and are considered potential markers in LMS. Accordingly, several HDACis have been used and tested alone or in combination with other drugs (Table 3). Vorinostat or valproic acid exhibited growth suppression of the MES-SA cells [130,131,132]. In a recently published study, tucidinostat, an HDAC inhibitor, increased the apoptosis process and inhibited the proliferation of LMS cells, which was proposed as a promising strategy for treating this aggressive uterine cancer [126]. Combination studies revealed that vorinostat with PI3K/AKT/mTOR pathway inhibitors, rapamycin, or LY294002 demonstrated a synergistic effect on inhibiting MES-SA cell growth [104]. HDACi treatment can reverse chemoresistance, enhance chemotherapy-induced cytotoxicity, and inhibit sarcoma cell proliferation in vitro [133,134,135]. The effect of HDACis has been investigated in several clinical trials with limited impact. An open-label, multicenter, phase II trial assessed the safety, toxicity, and effectiveness of the combination therapy with an HDAC inhibitor, mocetinostat, together with gemcitabine in a group of patients with metastatic LMS who had previously progressed during chemotherapy containing gemcitabine (NCT02303262). The combination of mocetinostat and gemcitabine was generally well tolerated but with no effect on chemoresistance reversion [136]. Another study based on vorinostat treatment was closed prematurely in 2019 due to slow patient recruitment and complex acquisition of the study product (https://classic.clinicaltrials.gov/ct2/show/NCT03509207, accessed on 20 June 2024). Combining VPA with bevacizumab (targeting VEGF), gemcitabine, and docetaxel enhances responses and alters chemoresistance. The results confirmed the safety of those four compounds’ administration. However, patients with LMS presented only partial response to treatment [137]. 

LMS cells exhibit higher HAT1 expression than LM cells, which has also been correlated with poor survival outcomes [138]. Combination treatment with pazopanib, a multitargeted tyrosine kinase inhibitor, and hyperthermia, inhibited HAT1 expression in LMS cells, which was mediated by Clock suppression. In turn, reconstitution of the Clock protein rescued both HAT1 levels and HAT1-mediated histone acetylation. The Clock element of the HAT1 promoter has been proven to be crucial in pazopanib and hyperthermia-induced downregulation of HAT1. Decreased HAT1 expression inhibits the acetylation of residues K5 and K12 on histone H4 (H4K5 and H4K12), reducing gene transcription and inhibiting tumor growth [138]. 

Bromodomain-containing proteins (BRDs), as readers of acetylated lysine on histones, transduce regulatory signals, and the deregulation of BRDs is involved in many diseases, including benign LM [139,140] and cancer [141]. Bromodomain-containing protein 9 (BRD9) belongs to the chromatin remodeling/sucrose non-fermentable (SWI/SNF) switch complex, which interacts with chromatin and transcription factors involved in cell proliferation, apoptosis, differentiation, and cancer. The BRD-9 expression was upregulated in uLMS compared to myometrium, and targeting BRD9 with its inhibitor, TP-472, contributed to the induction of cell death and cell cycle arrest and was also involved in the modulation of the oncoepigenome in LMS cells [142]. In addition to an in vitro study on the role of BRD “reader” inhibition in LMS, a BET bromodomain inhibitor GS-626510 has been tested in the LMS PDX model harboring either derangements in C-MYC and PTEN/PI3CA/AKT genes or homologous recombination deficiency signatures. The study by Choi et al. showed that GS-626510 suppressed the LMS tumor growth in these two models [59]. These studies suggest the important role of histone readers in the pathogenesis of LMS (Figure 3, Table 3). 

### 4.3. Non-Coding RNA

Non-coding RNAs (ncRNAs) are involved in the altered transcription processes by modulating heterochromatin, histone modification, and DNA methylation [143]. Among several types of non-coding RNAs, intercellular miRNAs have been extensively investigated and demonstrated their important roles in cell signaling, proliferation, migration, and angiogenesis in LMS development [144] (Table 4). A few comparison studies between myometrium and LMS at tissue and cell levels identified many differentially expressed miRNAs [145,146,147,148,149]. Among them, the function of several miRNAs in uLMS has been characterized. miR-10b-5p significantly inhibited cell proliferation, reduced the number of colonies, and increased the number of uLMS cells in the G1 phase [145]. miR-130b promoted aggressiveness in LMS cells by accelerating tumor growth and enhancing metastatic potential [150]. The Let-7 family was involved in the risk of death in patients with uLMS and considered an independent prognostic factor [151]. miR-1 exhibited its antiproliferative and proapoptotic properties [152,153]. miR-1 was strongly suppressed in LMS tumor tissue, with expression levels decreasing 2.4-fold in samples from LMS patients compared to healthy donors. However, in vitro studies did not demonstrate growth inhibitory properties of miR-1 in uLMS cells [154]. One of the targets of miR-140-5p is IGFR1, the overexpression of which is associated with the inhibition of apoptosis and increased tumorigenic potential because of hindered programmed cell death [155]. miR-1-3p is considered to modulate critical genes such as Bcl-2 that regulate apoptosis [148]. One of the targets of miR-140-5p is IGFR1, the overexpression of which is associated with the inhibition of apoptosis and increased tumorigenic potential as a result of hindered programmed cell death. [155]. miR-152 is a molecule involved in proliferation, invasion, and angiogenesis [156,157]. miR-152 transfection in SK-LMS-1 cells reduced mRNA and protein levels of KIT and MET, receptor tyrosine kinases, involved in sarcoma development and progression [158]. 

A comparison of primary and metastatic LMS lesions showed lower miR-15a and miR-92a levels and higher miR-31 levels in primary LMS. Downregulation of miR-15 removes the repression of target molecules driving cell proliferation, including Bcl-2, cyclin D1, and Mcl-1, in different cancers. miR-31 was involved in the MAPK and the Wnt signaling pathway in uLMS [159].

The role of differentially expressed miRNAs between the responders and non-responders for the treatment has been investigated [160]. In the case of LMS, the three most abundantly expressed miRNAs were miR-106a, miR-17, and miR-34a, where oncogenic properties have already been proven for miR106a-363. When assessing the response to treatment, eribulin was found to be correlated with molecules belonging to the miR-17 family. In turn, miR-34 influenced metastasis and modulated the cell response to chemotherapeutic agents, and high levels of miR-34a prevented treatment resistance [161]. It was found that altered miR-34a expression may sensitize soft tissue sarcoma to eribulin, a proapoptotic compound [160] (Figure 3, Table 4). 

### 4.4. RNA Methylation

RNA modifications go beyond the traditional four RNA residues, revealing a complex tapestry of chemical alterations contributing to the intricate regulation of multiple cellular processes. Among over 170 identified RNA modifications, N6-methyladenosine (m^6^A), methylated at the N6 position of adenosine, is abundant, reversible, and modulated by methyltransferases (writers) and demethylases (erasers). In addition, different “readers” can recognize and bind to the m^6^A-modified RNAs, including mRNAs and non-coding RNAs, and alter their fate [162,163]. m^6^A RNA modification can stabilize RNAs and regulate their localization, transport, and post-translational regulation, therefore controlling multiple cellular processes. The m^6^A modification is catalyzed by methyltransferases including methyltransferase-like 3 (METTL3), METTL14, and their cofactors such as WTAP, VIRMA, and RBM15 [164,165,166]. The fat mass and obesity-associated protein (FTO) and AlkB homologue 5 (ALKBH5), as demethylases, can remove the m^6^A modification [167,168,169]. The m^6^A RNA modification can impact post-transcriptional gene expression through specific recognition by m^6^A-binding proteins, such as YTHDF1/2/3, YTHDC1/2, and IGF2BP1-3 [170,171,172]. 

Abnormal expression of m^6^A regulators has been shown to be involved in many pathological events, including tumorigenesis [173,174,175,176]. We recently demonstrated that FTO and ALKBH5 RNA demethylases were aberrantly upregulated in uLMS compared to adjacent myometrium by immunohistochemistry analysis. A small, potent FTO inhibitor, DAC51, remarkably suppressed the uLMS proliferation by inducing cell cycle arrest. Unbiased analysis of high-throughput RNA sequencing identified the enrichment of several critical pathways, including the G_2_M checkpoint, C-MYC signaling, TNFα signaling, inflammation response, and KRAS signaling, in response to DAC51 treatment, in alignment with the uLMS phenotype alteration [177]. Additionally, the intersection of RNA methylation and other epigenetic mechanisms via FTO inhibition has been characterized. Therefore, targeting the vulnerable RNA methylation machinery may provide a promising and novel option for treating patients with this aggressive uterine cancer (Figure 3). 

These findings suggest that multiple epigenetic mechanisms are involved in the pathogenesis of uLMS. Therefore, targeted inhibition of epigenetic modulators may provide a promising and novel strategy for treating patients with this aggressive disease. 

## 5. Treatment of Uterine Leiomyosarcoma

The management of uLMS remains challenging due to its aggressive behavior. Although targeted treatments have increased the options for treatment for subpopulations, the overall risk for recurrence and mortality is high for women with this disease [106,178,179]. Surgery is an important aspect of treatment as primary surgical resection is the standard of care for patients with resectable disease. Although preoperative diagnosis may be challenging to obtain, imaging and endometrial biopsy prior to surgery for those with clinically suspicious disease may help provide appropriate surgical intervention and counseling. 

### 5.1. Surgery Management

According to the National Comprehensive Cancer Network (NCCN) guidelines, surgical resection should be performed first in medically operable patients. This should include a hysterectomy with or without bilateral salpingo-oophorectomy (BSO) and en-bloc resection of the tumor [180]. Premenopausal women may choose to retain their ovaries due to the lack of evidence to support a difference in OS among patients who underwent BSO compared to those who did not [25,181,182,183]. Furthermore, routine lymph node dissection is not recommended. uLMS tends to metastasize hematogenously [183], which is supported by the low incidence of lymph node metastasis (<5%) in early clinical stage disease [52]. Regardless, notably enlarged lymph nodes should be surgically removed. Patients who undergo optimal cytoreductive surgery with no gross residual disease may demonstrate a better outcome compared to those with suboptimal tumor debulking surgery [26,182,184,185,186]. Although residual disease is associated with a worse prognosis, surgery may still be considered in those for which hysterectomy may provide palliation for symptoms such as vaginal pain or bleeding. The surgical stage ultimately determines prognosis and further adjuvant therapy recommendations. Stage I is limited to the uterus, stage II extends beyond the uterus into the pelvis, and stage III involves disease in the abdomen, while stage IV includes metastatic disease involvement organs such as the bladder, rectum, or distant metastatic sites. Survival is predicted by stage [180]. The 5-year disease-specific survival for patients with stage I is 76%, stage II 60%, stage III 45%, and stage IV 29% [181]. Although morphology has not been shown to predict clinical behavior, tumor size, and mitotic rate have been shown to be prognostic [187].

### 5.2. Adjuvant Therapy

Following a surgical diagnosis of uLMS, the use of adjuvant therapy is controversial. Currently, there is no evidence to support the use of adjuvant radiation. Although a phase III randomized European Organisation for Research and Treatment of Cancer (EORTC) study evaluating adjuvant radiotherapy in all uterine sarcoma subtypes showed an initial reduction in local relapse following adjuvant radiation compared to observation (14% vs. 24%, *p* < 0.01 in all subtypes, there was no difference in overall or disease-free survival in uLMS [188]. For patients with extra-uterine extension, radiation therapy is a reasonable consideration, but the role of adjuvant postoperative radiation remains limited. Additionally, for patients with early-stage disease, stage I or II, there is little evidence to support the use of systemic adjuvant treatment such as chemotherapy [189,190]. GOG-0277, a two-arm open-label randomized phase III trial evaluating adjuvant chemotherapy with gemcitabine combined with docetaxel followed by doxorubicin versus observation in women with stage I uLMS failed to accrue their target of 216 patients [191]. However, for the 38 enrolled patients, the restricted mean survival time for OS was 34.3 mo in the chemotherapy arm compared to 46.4 mo in the observation arm. The restricted mean survival time for recurrence-free survival was estimated to be 18.1 vs. 14.6 months in the chemotherapy and observation arms, respectively. Although the sample size did not allow for adequate statistical analysis, the observed survival data showed no superior outcomes for adjuvant therapy. Ultimately, no adjuvant therapy is recommended for stage I disease per NCCN guidelines. 

### 5.3. Primary Systemic Treatment 

For patients with metastatic or unresectable disease, systemic treatment should be considered for primary treatment. Anthracycline-based combination regimens can be used for uterine sarcomas including uLMS [180] with good response rates, but none have demonstrated improved survival. Doxorubicin has a dose–response relationship, with poor efficacy for doses lower than 60 mg/m^2^, and instead, a dose of 75 mg/m^2^ is recommended [192]. 

A phase III multi-center EORTC study evaluated single-agent doxorubicin use (75 mg/m^2^) versus doxorubicin combined with ifosfamide (10 g/m^2^) (AI) in advanced-stage soft tissue sarcomas [193]. Although combination therapy with AI demonstrated a higher response rate (26.5% vs. 13.6%, *p* < 0.05) and improved PFS (7.4 mo vs. 4.6 mo HR 0.74, *p* < 0.05), there was no significant difference between the two regimens in OS (14.3 mo vs. 12.8 mo, HR 0.83, *p* = 0.076). Higher levels of toxicity for the AI group also made completing six cycles of therapy difficult. 

Gemcitabine combined with docetaxel is a commonly utilized regimen for primary systemic treatment for metastatic or non-operable uLMS. In a phase II trial in the front-line setting, gemcitabine (900 mg/m^2^) combined with docetaxel (100 mg/m^2^) demonstrated an overall response rate of 35.8% [194]. The median PFS was 4.4 months while the median OS was over 16 months. However, in the phase 3 GeDDiS trial, doxorubicin alone had similar PFS and OS compared to gemcitabine plus docetaxel use (PFS 23.3 weeks vs. 23.7 weeks, HR 1.26, *p* = 0.08) (OS 76.3 weeks vs. 67.3 weeks, HR 1.14 *p* = 0.41) in all soft-tissue sarcomas, including uLMS [195].

Currently, the NCCN recommends a combination of trabectedin, a DNA-binding agent, with doxorubicin as the first line of therapy for metastatic or non-operable uLMS [180]. In a randomized phase III superiority trial, single-agent doxorubicin (75 mg/m^2^) was compared to doxorubicin (60 mg/m^2^) combined with trabectedin (1.1 mg/m^2^) followed by maintenance trabectedin alone [196]. The combination of trabectedin with doxorubicin provided a significantly longer PFS (12.2 vs. 6.2 mo; HR 0.41; *p* < 0.0001) [196]. However, the toxicities were higher in the combination group. The most common grade 3–4 toxicities included neutropenia (80% vs. 13%), anemia (31 vs. 5%), thrombocytopenia (47 vs. 0%), and febrile neutropenia (28 vs. 9%). Thus, caution must be given when selecting appropriate patients for this regimen (Table 5).

### 5.4. Systemic Treatment for Recurrent Disease

For recurrent disease, a few chemotherapies have been found to be effective in the second-line setting. As mentioned above, the combination of gemcitabine and docetaxel has an ORR of 27% in the recurrent setting [197], even after having received doxorubicin. Overall, 52% of patients were progression-free at 6 months. Single-agent doxorubicin may be considered as a second-line option if the patient did not receive front-line doxorubicin-based treatment. 

Trabectedin can be utilized in the second-line treatment of uLMS. Although in the single-arm phase II trial, there was an ORR of 59.6% [198], and the randomized phase II trial showed no difference in response rate or PFS compared to doxorubicin alone [199]. In a multi-center phase III trial evaluating trabectedin (1.5 mg/m^2^) versus standard of care dacarbazine (1 g/m^2^) in patients with advanced liposarcoma or uLMS after prior treatment with an anthracycline and at least one other agent, patients receiving trabectedin had an improved PFS (4.2 mo vs. 1.5 mo, HR 0.55, *p* < 0.001) [200]. Additionally, the clinical benefit was significantly higher in the trabectedin group (34% vs. 19%, *p* < 0.001). Thus, trabectedin can be used for advanced uLMS after progression following anthracycline-based treatment. Pazopanib (800 mg daily) is an oral multi-kinase inhibitor that blocks VEGFR, PDGFR, FGFR, and c-kit. It is currently approved in the USA for metastatic soft tissue sarcomas following progression after anthracycline treatment. In a randomized multi-center phase III trial comparing pazopanib against a placebo, pazopanib showed an improved median PFS of 4.6 months compared to 1.6 (HR 0.31, *p* < 0.0001), but no significant difference in median OS (12.5 mo vs. 10.7 mo, HR 0.86, *p* = 0.25) [201]. The ORR was 6%. 

Eribulin is a synthetic analog of halichondrin B and a microtubulin inhibitor, used as second-line therapy in uLMS, otherwise also indicated for the treatment of breast cancer. A randomized open-label phase III clinical trial compared eribulin (1.4 mg/m^2^) to dacarbazine (850 mg/m^2^, 1000 mg/m^2^, or 1200 mg/m^2^) for patients with LMS or adipocytic sarcoma with at least two prior lines of treatment [202]. Patients who received eribulin had an improved OS (13.5 mo vs. 11.5 mo, HR 0.77, *p* = 0.0169). However, this benefit may have been restricted to the liposarcoma patients only, as OS was not superior in the LMS subgroup (12.8 vs. 12.3 mo). The PFS for the LMS subgroup was 2.2 months compared to 2.6 months with dacarbazine. Thus, dacarbazine may be more active in LMS than liposarcoma (Table 5). 

NCCN guidelines also offer biomarker-directed therapies as second-line treatment options. For metastatic or non-resectable tumor mutational burden (TMB)-high tumors (≥10 mut/MB), pembrolizumab is offered as an option, given its site-agnostic indication. For patients with tumors harboring an NTRK fusion, Larotrectinib or entrectinib may be offered. For BRCA-altered LMS, PARP inhibitors may be an alternative [64,203,204]. In a single-arm, open-label, multicenter phase II trial evaluating olaparib (200 mg orally twice a day) and temozolomide (75 mg/m^2^ orally once daily) in advanced-stage uLMS with 55 patients, the overall response rate was 27% with a median PFS of 6.9 months. An exploratory analysis showed that median PFS was longer for homologous recombination-deficient (HRD) patients compared to HR-proficient patients (11.2 vs. 5.4 months *p* = 0.05) [205]. These studies reveal that in addition to surgery, radiation therapy is reversed for selected cases. Systemic treatment and active chemotherapy regimens are recommended for advanced-stage patients with uLMS.

## 6. Future Perspective

### 6.1. Clinical Diagnosis between Malignant Uterine Leiomyosarcoma and Benign Leiomyoma 

#### 6.1.1. Serum Biomarkers

Human serum biomarkers have been successfully used as an adjunct in the diagnosis of many diseases, including ovarian cancer. The most efficient biological diagnostic tool for ovarian cancer is the combination of Carbohydrate Antigen 125 (CA125) and Human Epididymis Protein (HE4). The use of these biomarkers together or as part of an algorithm (risk of malignancy index (RMI) or risk of ovarian malignancy algorithm (ROMA)) has led to a paradigm shift in the preoperative diagnosis of ovarian cancer [206]. It is important to acknowledge that developing effective serum biomarkers is an overwhelmingly challenging task. Even in the case of ovarian cancer, a validated screening strategy for use in the general population has yet to be developed [207]. Despite this, it is understood that the utilization of serum biomarkers as an adjunct to individual patient factors, epidemiologic data, and radiologic modalities is highly beneficial in distinguishing benign from malignant lesions preoperatively [206]. As a result, much work has been and continues to be carried out by our team [53,208] and others worldwide [209] to develop new, meticulously evaluated research strategies to identify serum biomarkers to distinguish LM from uLMS. The identification of such serum biomarkers that are differentially expressed in uLMS as compared to LM has the potential to transform the preoperative distinction of uLMS from LM.

It has been recently reported that m^6^A is actively released in response to cytotoxic stimulation and that extracellular m^6^A facilitates its pathological progress, which is different from that of adenosine [210]. m^6^A is an endogenous A3 adenosine receptor ligand. Understanding the role of extracellular modified nucleotides in distinguishing uLMS vs. LM is worthwhile. 

#### 6.1.2. Advanced Imaging—Artificial Intelligence and Machine Learning

Radiomics is the extraction of mineable data, i.e., quantitative features from radiologic images using computer algorithms. Artificial intelligence (AI) combined with machine learning improves diagnostics, prognostication, and clinical decisions [211]. Within women’s health, AI has primarily been used in breast imaging. In contrast, gynecologic imaging has been underserved in the field of AI. A systematic review by Shrestha et al. [211] reports that AI in gynecology, though limited, has most frequently been used in endometrial, cervical, and ovarian cancers. MRI is the imaging modality most utilized for AI in these cancers, followed by CT scans and ultrasounds. The authors conclude that despite some of the limitations of AI in gynecology, including generalizability and reproducibility concerns, AI shows promise for use in gynecology. Specifically, within the context of uterine tumors, AI could be a powerful tool in differentiating LM from uLMS.

#### 6.1.3. Shear Wave Elastography

We previously described the morphology of LM and uLMS. The former classically appears as a well-circumscribed, firm, and rubbery tissue with a whorled appearance, while sarcomas tend to have a softer and more necrotic texture or consistency [51]. Studies have demonstrated that tissue stiffness directly affects tumor growth through the induction of fibrosis [212,213,214]. Fibrosis, in turn, affects cell growth rate through a process called mechanotransduction in which mechanical forces or stiffness applied to cells are translated into chemical signaling that results in adaptive responses [46,212]. Since tissue stiffness intimately interplays with tumor cell signal transduction and tumor growth, it is possible that tumor stiffness may correlate with tumor behavior.

The ultrasound-based technology, shear wave elastography (SWE), is an objective, quantitative technique to provide color elastograms with anatomic specificity. With this characteristic, SWE can assess the tissue stiffness to give information on tissue characterization. SWE has been validated against traditional material testing techniques [215,216] and has successfully been incorporated into standard ultrasound exams. This technology is currently being used as a standard practice in the management of liver cirrhosis, breast masses [217], and incompetent cervix [218]. Only a few reports of SWE use in gynecology and specifically for LM have been published with encouraging results [219,220,221]. With our increased understanding of the link between tissue morphology and tumor behavior, the successful incorporation of SWE into gynecological practice may help determine the severity of uterine diseases and treatment response. 

### 6.2. Prevention 

The FDA has approved several agents for premalignant disease or cancer prevention [222]. The diseases include breast cancer, Barrett’s esophagus, bladder cancer, skin cancer, cancers associated with HPV infection, and cancers related to HBV infection [222]. For example, tamoxifen and Raloxifene were used to reduce the incidence or risk of breast cancer in women [223,224,225,226]. Currently, knowledge about the risk factors of uLMS is limited. Therefore, understanding the origin of uLMS and establishing a relevant experimental model will help identify agents with a preventative effect on developing this aggressive uLMS.

**Table 3:** Drugs targeting histone modifications in uterine sarcoma cells.

Drugs/Factors	Epigenetic Targets	Biological Samples	Effectors	Biological Effect	Approach	Publication Time	Refs.
Vorinostat	HDACs	MES-SA	p21	apoptosis, tumor growth inhibition	in vitro and in vivo	2010	[131]
Valproic acid	HDACs	MES-SA	NA	cytotoxic effect	in vitro	2013	[130]
SAHA, LY294002, rapamycin	HDACs	MES-SA	AKT, mTOR/p70S6K	growth inhibition	in vitro	2014	[104]
FASN	H3K9me3, H3K27ac	SK-UT-1	CRISP1	proliferation, migration, cellular motion	in vitro	2017	[227]
GS-626510	BET bromodomain	uLMS PDX model	NA	tumor growth inhibition	in vivo	2021	[59]
Tucidinostat, sulforaphane	HDACs	SK-UT-1	PCNA, CDK members	cell proliferation,	in vitro	2022	[126]
TP-472	BRD9	SK-UT-1	H3K4me3, YTHDC1, YTHDF2	cell cycle, cell proliferation, apoptosis	in vitro	2022	[142]

Note: LY294002: inhibitor of PI3K; rapamycin: mammalian target of rapamycin mTOR; FASN: fatty acid synthase; NA: not available; LM: leiomyoma.

**Table 4:** miRNAs involved in the pathogenesis of uterine leiomyosarcoma.

MiRNAs	Biological Samples	Expression	Targets	Biological Events	Year Published	Refs.
let-7s	LMS tissues, SK-LMS-1, SK-UT-1, and SK-UT-1B cell lines	downregulated	*HMGA2*	cell proliferation	2008	[228]
72 miRNAs	LMS tissues	deregulated (32 up-Mir, 40 down-Mir)	NA	differentiation, neoplastic transformation	2010	[146]
miR-200c	SK-LMS-1	downregulated	*IKBKB*, *IL8*, *CDK2*, and *CCNE2*	inflammatory response, angiogenesis, cell cycle, migration	2014	[149]
miR-31	metastatic and primary LMS tissues, SK-LMS-1	downregulated in metastatic LMS tissues	MAPK signaling, Wnt signaling	metastasis	2016	[159]
miR-15a, miR- 92a	metastatic and primary LMS tissues	upregulated in metastatic LMS tissues	Wnt signaling	metastasis	2016	[159]
13 miRNAs	LMS, MM, LMS-derived cell line	deregulated (8 up-Mir, 5 down-Mir)	*BCL2, EGFR, VEGFA, IGF1R, EGF-R, MET, MYCN*	tumor apoptosis, angiogenesis, proliferation	2017	[147]
miR-152	LMS tissues, SK-LMS-1	downregulated	*MET*, *KIT,* PI3K/AKT (transcription factors)	cell proliferation, apoptosis, cell cycle	2017	[229]
miR-1	LMS tissues	downregulated	NA	disrupted tumor suppression	2018	[154]
let-7 family	LMS tissues	downregulated	NA	PFS, OS	2019	[151]
miR-1246, miR-191-5p	serum from LMS patients	downregulated	NA	diagnostic biomarker	2019	[230]
miR-10b-5p	LMS tissues, SK-UT-1, SK-LMS-1	downregulated	G1/S checkpoint, MYC-mediated apoptosis, epithelial–mesenchymal transition	cell proliferation, cell cycle	2023	[145]
miR-130b	LMS, MM tissues, SK-LMS-1, SK-UT-1	upregulated	*TSC1*	tumor proliferation and metastasis	2023	[150]

Note: LMS: leiomyosarcoma; MM: myometrium; NA: not available; PFS: progression-free survival; OS: overall survival.

**Table 5:** Clinical results targeting uterine leiomyosarcoma.

Drug(s) and Clinical Trial Identifier	Study Design	Indication	Grade 3 and 4 Toxicities	Efficacy	Year	Refs.
Intensified doxorubicin plus ifosfamide NCT00061984 EORTC 62012 (complete)	EORTC phase 3 randomized study evaluating OS of intensified doxorubicin plus ifosfamide vs. doxorubicin use as first-line treatment	locally advanced, unresectable, or metastatic high-grade soft tissue sarcoma and no prior systemic cytotoxic treatment (but adjuvant chemo allowed)	ebrile neutropenia (46%) leukopenia (43%) neutropenia (42%) anemia (35%) thrombocytopenia (33%)	mPFS: 7.4 mo vs. 4.6 mo HR 0.74 *p* = 0.003 mOS: 14.3 mo vs. 12.8 mo HR 0.83 *p* = 0.076 ORR: 26% (60/227)	2014	[193]
Fixed-dose rate gemcitabine plus docetaxel	GOG phase 2 study evaluating PFS of fixed-dose rate gemcitabine plus docetaxel use as first-line treatment	metastatic unresectable uLMS and no prior systemic cytotoxic treatment	anemia (23.8%) thrombocytopenia (19%) neutropenia (16.7%) fatigue (16.7%) metabolic toxicities (16.7%) leukopenia (14.3%) GI toxicity (14.3%)	mPFS: 4.4 mo mOS: 16.1+ mo ORR: 35.7% (15/42)	2008	[194]
Doxorubicin plus trabectedin NCT02997358 (Complete)	randomized phase 3 study evaluating PFS of doxorubicin and trabectedin use vs. doxorubicin alone as first-line treatment	metastatic or relapsed unresectable LMS without prior systemic treatment	neutropenia (80%) leukopenia (75%) thrombocytopenia (57%) ALT increase (42%) anemia (31%) renal creatinine clearance decreases (31%) febrile neutropenia (28%) fatigue (11%)	mPFS: 12.2 mo vs. 6.2 mo HR 0.41, *p* < 0.0001 ORR: 36% (27/74) vs. 13% (10/74) uLMS ORR: 36% (12/33) vs. 15% (5/34)	2022	[196]
Fixed-dose rate gemcitabine with docetaxel	GOG phase 2 study evaluating efficacy of fixed-dose gemcitabine with docetaxel	advanced or recurrent uLMS progressed after at least one prior line excluding gemcitabine or docetaxel use	thrombocytopenia (39.5%) anemia (25%) leukopenia (23%) neutropenia (20.8%)	mPFS: 6.7+ mo mOS: 14.7 mo ORR: 27% (13/48)	2008	[194]
Trabectedin NCT01343277 (Complete)	randomized phase 3 study evaluating OS of trabectedin use compared to dacarbazine use	advanced liposarcoma or LMS after at least two prior lines with at least one containing anthracycline	neutropenia (37%) ALT elevation (26%) thrombocytopenia (17%) anemia (14%) AST elevation (13%)	mPFS: 4.2 mo vs. 1.5 mo HR 0.55, *p* < 0.001 mOS: 12.4 mo vs. 12.9 mo HR 0.87, *p* = 0.37 ORR: 9.9% (34/345) vs. 6.9% (12/173), *p* = 0.33	2016	[200]
Pazopanib NCT00753688 (Complete)	randomized phase 3 study evaluating PFS of pazopanib use compared to placebo	progressive metastatic soft tissue sarcoma with at least one prior line containing anthracycline, up to four prior lines	fatigue (14%) hypertension (7%) anorexia (6%)	mPFS: 4.6 mo vs. 1.6 mo HR 0.31, *p* < 0.0001 mOS: 12.5 mo vs. 10.7 mo HR 0.86, *p* = 0.2514 ORR: 14/246 (6%) vs. 0/123 (0%)	2012	[201]
Eribulin NCT01327885 (Complete)	randomized phase 3 study evaluating OS of eribulin compared to dacarbazine use	Intermediate- or high-grade advanced-stage liposarcoma or LMS with at least two prior lines including anthracycline use	neutropenia (35%) leukopenia (10%) anemia (7%)	mPFS: 2.2 mo vs. 2.6 mo HR 1.07, *p* = 0.58 mOS: 13.5 mo vs. 11.5 mo HR 0.77, *p* = 0.0169 LMS subgroup mPFS: 1.4 vs. 2.6 mo, HR 1.57 mOS: 12.7 mo vs. 13.0 mo HR 0.93 ORR 9/228 (4%) vs. 11/224 (5%)	2016	[202]
Olaparib and temozolomide NCT03880019 (Complete)	phase 2 single-arm open-label study evaluating olaparib and temozolomide	advanced and unresectable or metastatic uterine LMS patients	neutropenia (75%) thrombocytopenia (32%) leukopenia (22%)	mPFS 11.2 mo in HR-deficient patients vs. mPFS 5.4 mo in HR-proficient patients *p* = 0.05 ORR 6/22 (27%)	2023	[205]
Olaparib and temozolomide NCT05432791 (Active)	randomized phase 2/3 study evaluating PFS and OS of olaparib plus temozolomide compared to investigator’s choice	advanced and unresectable or metastatic uLMS patients who received two or more prior lines including anthracycline use	NA	NA	ongoing	NA
Lurbinectedin and doxorubicin NCT05099666 (Active)	phase Ib/2 study exploring safety and efficacy of lurbinectedin and doxorubicin	Phase Ib: advanced or metastatic soft-tissue sarcoma with no more than two prior lines, and no prior anthracycline or trabectedin use. Phase 2: advanced or metastatic LMS with no more than one prior line and no prior anthracycline or trabectedin use.	NA	NA	ongoing	NA
Gemcitabine, dacarbazine, and HIPEC NCT04727242 (Active)	phase 2 study evaluating the use of Cytoreductive Surgery and Hyperthermic Intraperitoneal Chemotherapy (HIPEC) with gemcitabine followed by systemic adjuvant chemotherapy with dacarbazine	locally recurrent uLMS without extra-abdominal disease, and no prior gemcitabine or dacarbazine use	NA	NA	ongoing	NA

Abbreviations: NA: not available; PFS: progression-free survival; OS: overall survival.

### 6.3. Future Clinical Trials

Additional therapeutics are currently being investigated for their efficacy in treating uLMS in the recurrent setting. NCT05432791 is a phase II/III clinical trial evaluating the combination of olaparib with temozolomide compared to the investigator’s choice for the treatment of patients with advanced uLMS for those who had at least two prior lines of therapy, regardless of BRCA or HRD status. Lurbinectedin is a synthetic alkylating agent used in small-cell lung cancer, which is currently being investigated in a phase Ib/II trial in combination with doxorubicin in advanced and metastatic soft tissue sarcoma or LMS (NCT05099666). NCT04727242 is a phase II trial evaluating the efficacy of cytoreductive surgery with gemcitabine hyperthermic intraperitoneal chemotherapy (HIPEC) followed by adjuvant dacarbazine in locally recurrent uLMS. Although adjuvant systemic therapy is not effective in uterine-limited disease, this may be further investigated in the future.

## Figures and Tables

**Table 1:** Comparison between leiomyoma and uterine leiomyosarcoma.

Features	Uterine Leiomyoma	Uterine Leiomyosarcoma
**Clinicopathologic**	*Similarities* Abnormal uterine bleeding Pelvic pressure Pelvic mass *Differences* No growth after menopause in the absence of hormonal therapy Commonly exists in multiples	*Similarities* Abnormal uterine bleeding Pelvic pressure Pelvic mass *Differences* Growth after menopause Commonly exists as a solitary mass
**Tissue Origin**	*Similarities* Originates in the myometrium of the uterus *Differences* Cell type: MMSCs	*Similarities* Originates in the myometrium of the uterus. *Differences* Cell type: unknown
**Genetic Abnormality**	*MED12* and *HMGA2* mutation	Complex karyotypes and aneuploids
**Morphology**	*Similarities* May be a solitary uterine tumor Express progesterone, estrogen, and androgen receptors Myxoid features of degenerated LM can be seen in LMS *Differences* Sharply demarcated or defined border Variants may exhibit increased cellularity and infarct-type necrosis	*Similarities* May be a solitary uterine tumor Express progesterone, estrogen, and androgen receptors Myxoid features of degenerated LM can be seen in LMS *Differences* Invades adjacent myometrium Extensive areas of tumor cell necrosis and hemorrhage, hypercellularity, and severely atypical nuclei with >15 mitotic figures per 10 high-power fields
**Behavior, Onset, Incidence**	*Similarities* Incidence increases with age Common at age >40 More prevalent in Black women *Differences* Common and benign More common in reproductive age No growth after menopause Low mortality rate May be asymptomatic	*Similarities* Incidence increases with age Common at age >40 More prevalent in Black women *Differences* Rare and malignant More common in late perimenopause and menopause Growth after menopause High mortality rate Rarely asymptomatic

Note: LM: leiomyoma; LMS: leiomyosarcoma.

**Table 2:** Comparison of histological features among conventional LMS, epithelioid LMS, and myxoid LMS.

Histologic Features	Conventional LMS	Epithelioid LMS	Myxoid LMS
**Essential diagnostic criteria**			
	(2 of 3 histologic features):	(≥1 feature):	(≥1 feature):
Cytologic Atypia	Severe	Moderate to severe	Moderate to severe
Necrosis	Present	Present	Coagulative necrosis
Mitosis	≥10 mitoses/10 high power fields	often about 3 mitoses/10 high power fields	often about 3 mitosis/10 high power fields
Margins	infiltrative border	infiltrative borders	infiltrative borders
**Cytologic features**			
Cell type	Spindle/elongated	>50% round or polygonal	
Cytoplasm	Eosinophilic	Eosinophilic, extensive hyalinization	scant
Nucleus	Hyperchromatic/pleomorphic Multinucleated Atypical mitoses	mild nuclear atypia	nuclear pleomorphism
**Growth pattern**			
	Cellular tumor comprised of long intersecting or haphazard fascicles	Arranged in nests, cords, or sheets May show pseudo-glandular spaces	Hypocellular tumor with abundant myxoid stroma Myxoid stroma may be difficult to differentiate from hydropic change in small/limited samples

## Data Availability

Not applicable.
